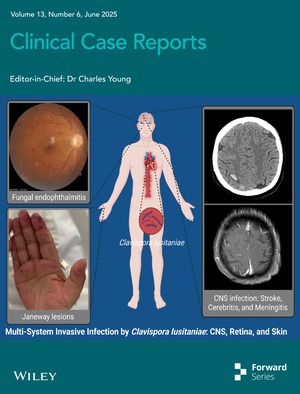# Cover Image

**DOI:** 10.1002/ccr3.70572

**Published:** 2025-06-16

**Authors:** Yi Cui, Zhenxing Huang, Hui Han, Yang Ma, Shanshan Li, Yuanyuan Hu, Hui Zhang, Xuehai Zhang, Xiuhe Zhao, Dexin Yu, Jianqiao Li, Han Liu, Chen Li, Hao Wang

## Abstract

The cover image is based on the article *Encephalopathy and Endophthalmitis Following Device‐Related Invasive Clavispora Lusitaniae Infection in an Immunocompetent Patient: A Case Report* by Hao Wang et al., https://doi.org/10.1002/ccr3.70550.